# Participatory definition of breeding objectives for sheep breeds under pastoral systems—the case of Red Maasai and Dorper sheep in Kenya

**DOI:** 10.1007/s11250-015-0911-7

**Published:** 2015-09-15

**Authors:** Emelie Zonabend König, Tadele Mirkena, Erling Strandberg, James Audho, Julie Ojango, Birgitta Malmfors, Ally Mwai Okeyo, Jan Philipsson

**Affiliations:** Department of Animal Breeding and Genetics, Swedish University of Agricultural Sciences, P.O. Box 7023, 750 07 Uppsala, Sweden; International Livestock Research Institute, Nairobi, Kenya

**Keywords:** Participatory approach, Livestock farmers, Breeding goals, Crossbreeding, Endangered breed

## Abstract

Crossing local breeds with exotic breeds may be an option for increased livestock productivity. However, there is a risk for endangerment of the local breeds. One such case is in Kenya where the imported Dorper breed is used for crossbreeding with Red Maasai sheep. The aim of this study was to investigate farmers’ trait preferences as a basis for determination of breeding objectives for Red Maasai and Dorper sheep at two sites, Amboseli and Isinya, in Kenya. Within their own flock, each farmer identified three ewes representing the best, average and poorest within each breed group: Red Maasai, Dorper and Crosses. Farmers gave reasons for their ranking. Body measurements and weights were also taken. At the harshest site, Amboseli, differences between breed groups in body weight were small and breeds were equally preferred. In Isinya, where environmental conditions are better and farmers are more market oriented, Dorper and Crosses had significantly higher body weights and market prices and were thus preferred by the farmers. Red Maasai were preferred for their maternal and adaptive traits. Breeding objectives should emphasize growth traits and milk production in both breeds at both sites. Body condition needs to be specifically considered in the breeding objectives for sheep in Amboseli, whereas adaptive traits need to be generally emphasized in Dorper.

## Introduction

A number of livestock production systems in developing countries and low-input systems are under intensification as shown in a review by Marshall (2014). Exotic breeds are often introduced and used for crossbreeding with local, indigenous breeds to increase the productivity. Very little research has, however, been conducted on optimal use of different breed types (Marshall 2014). There is a risk that the exotic breeds are used too extensively and without a long-term plan although they may not be adapted to harsh environments and recurrent droughts and diseases.

Red Maasai, Dorper and their crosses are examples of important sheep breeds for the livelihood of people in Kenya and Tanzania (Marshall et al. 2013). The Red Maasai sheep is an East African fat-tailed sheep mainly kept by Maasai pastoralists and neighbouring tribes (Wilson 1991). The breed is renowned for its resistance against endoparasites (Preston and Allonby 1979; Baker et al. 1999; Silva et al. 2012) and relatively good tolerance to trypanosomes (Baker 1995) and drought (Kosgey 2004). The Red Maasai has, however, ranked poorly in terms of body weight relative to the Dorper and crosses (Kosgey 2004). The Dorper sheep was first introduced to Kenya from South Africa in 1952 for its growth potential, better carcass quality and mothering ability (Kiriro 1994; Kosgey et al. 2008; Kariuki et al. 2010). It is a synthetic meat-type breed created from the Dorset and Black Head Persian breeds (Baker et al. 1999; Milne 2000).

Until the mid-1970s, purebred Red Maasai sheep were the type of sheep kept in most of the southern pastoral lands of Kenya, probably numbering several million heads. In the 1970s, however, a large population of Dorper sheep was imported to Kenya for research and multiplication purposes. This resulted in widespread indiscriminate crossbreeding between Red Maasai and Dorper to achieve an increase in weight and body size. The upgrading towards the Dorper later proved unsuitable due to their poor survival rates in areas with a harsh environment (Gibson and Pullin 2005). Even though the World Watch List does not include the Red Maasai among threatened breeds, because of the inability of the system to track dilution, it is evident that the Red Maasai is clearly threatened (Gibson 2007).

East Africa suffered from a severe drought in 2008 and 2009, and millions of livestock died. Interviews with Maasai farmers revealed that during the drought the Red Maasai sheep survived better compared with Dorper sheep and higher grade Dorper crosses (Liljestrand 2012). A comparative study involving the same breeds at the International Livestock Research Institute ranch at Kapiti Plains Estate confirmed the higher survival rate of Red Maasai compared with Dorper and their crosses (Ojango et al. 2013). Similar results were reported by Okeyo and Baker (2005). So far, most studies have focused on data from research stations and not from village flocks (Gizaw et al. 2014).

Due to the valuable characteristics of the Red Maasai, specifically to tolerate drought and diseases, there is a need to conserve the pure breed by implementing a breeding strategy that ensures its continued genetic improvement. Moreover, a controlled crossbreeding programme involving the two breeds, Red Maasai and Dorper, may be needed to combine the strengths of the two breeds and to sustainably improve livelihoods of their keepers. It is then necessary to ensure that farmers participate in the process of defining the breeding objectives (Wurzinger et al. 2011).

Participatory approaches of designing breeding objectives have been described previously (Kosgey 2004; Duguma et al. 2010). To succeed, it is necessary to consider the whole production system and involve stakeholders at every stage in the planning and operation of the breeding programme, integrating traditional behaviour and values (Kosgey 2004). Such approaches have been used for mixed livestock and pastoral systems for definition of breeding objectives in goats (Gizaw et al. 2010; Gebreyesus et al. 2013) and in operation of breeding programmes in Ethiopia (Mirkena et al. 2012). Participatory approaches have also been used in determining objectives of keeping dairy goats and for studies of farming systems for small ruminants in Kenya (Kosgey et al. 2008; Bett et al. 2009b). When identifying traits of importance for a breeding programme, surveys using structured interviews with the livestock keepers have often been used, e.g. for Sahiwal cattle kept by Maasai pastoralists in Narok and Kaijado County, Kenya (Ilatsia et al. 2012) and for endemic ruminant livestock in West Africa (Ejlertsen et al. 2012).

The aim of this study was to phenotypically characterize the Red Maasai and Dorper sheep and their crosses in terms of important traits and also to determine farmers’ trait preferences to inform definition of breeding objectives for their sheep flocks. The study included two Maasai pastoralist sites in Kenya representing two different production systems.

## Materials and methods

### Study sites

Pastoral livestock keepers from two Maasai pastoralist sites, Amboseli and Isinya in Kenya, keeping Red Maasai, Dorper and Crosses between the two sheep breeds, were included in the study. Pastoralists at these sites are mainly from the Maasai ethnic group and with an increasing influx of people from other ethnic groups as pastoral practices change. Farming of ruminants is common, and most people keep cattle, sheep and goats (Liljestrand 2012). The most southern site, Amboseli, includes the Selengei and Lenkisem villages. It is characterized by arid climate (Kenyan agro-ecozone V and VI) with little or no pastures during certain periods of the year and has small sized flocks of mixed livestock. There are no permanent rivers in the area. Farmers in Amboseli live in houses made of clay and mud and move their animals over large rangelands. In Isinya, on the other hand, pastures are more productive. Farmers have more permanent homes and move the animals only if the pasture availability is too low. Off-farm jobs are common in Isinya because it is close to the market and main urban centres. The sheep production is more market oriented and flock sizes are larger.

### Study design and data collection

Through a project that started 2 years ahead of this study, a basic sheep recording scheme was introduced to and supported by participating farmers. The scheme involved routine measurements of live weight and body size of individual animals with the aim of providing objective information to strengthen the farmers’ own qualitative valuation of important traits in their sheep to be used for breeding and management purposes.

Farmers included in this study were those identified by the communities at the sites as individuals keen on improving their sheep flocks. This was determined through focus group discussions and using key informants within the target areas (including local leaders, ministry of livestock development officials and NGOs). In addition, the sites were visited beforehand to ensure that sheep flocks existed and had a diversity of breed types. The selected farmers had, as far as possible, all three breed groups, i.e. Red Maasai, Dorper and Crosses. In total, 19 farmers were selected, ten from Amboseli and nine from Isinya. Of the 19 farmers, 11 had sheep from all breed groups, whereas six had only Red Maasai and Crosses and two had only Dorper and Crosses. Flock sizes ranged from 23 to 158 sheep in Amboseli and from 49 to 850 sheep in Isinya.

Farmers were requested to classify their sheep into breed groups mainly based on morphology and coat colour and, when possible, use of their knowledge of the pedigree of each animal. Within each of the three breed groups (i.e. Red Maasai, Dorper and Crosses), each farmer was asked in advance to select three ewes which had lambed at least once, and that according to the farmer’s opinion represented the Best, Average and Poor quality ewes, respectively. Every farmer provided at least three reasons in order of importance for their ranking of each ewe. In addition, they narrated the life history of the ewe, and what price (in Kenya Shillings; 1 USD = 84 KES) they would be willing to pay for the specific ewe if it was to be purchased at the market and to be used for breeding purposes. A local enumerator translated the open questions and answers directly into English. The answers given by the farmers about reasons for ranking followed a traditionally used terminology and were later transcribed and grouped into broader categories.

After having ranked ewes within each breed group, each farmer was asked to rank all the nine ewes across breed groups on a scale from 1 (most preferred) to 9 (least preferred). In cases of farms with only six ewes (two breed groups), results were re-scaled to correspond to rank values between 1 and 9 (new rank = 1 + (old rank-1) × 1.6). The objective was to get an overall view of the breed preferences by the farmers at the two sites.

Based on farmer’s recall, the pedigree (source of sire and dam) of each ewe was noted. An estimate of Milk Yield was obtained by asking the farmer how much milk a given ewe produces per day, after the lamb has suckled, in terms of number of cups. This was later translated into metric litres, by gradation of the volume of the cup used by each farmer. Additionally, for all ewes selected, linear body measurements, Body Weight, dentition (approximate age), Body Condition Score (BCS) and coat colour were recorded. Body Weight was taken for each ewe using a weigh scale provided to each farmer. Body Length was measured with tape from the shoulder joint to the hip joint. Heart Girth was measured around the chest, just behind the shoulder. BCS was assessed on a scale from 1 to 5 (half points were given), 1 being the poorest condition. A total of 147 adult ewes (51 Red Maasai, 39 Dorper and 57 crosses) belonging to the 19 farmers were characterized.

Descriptive statistics of the results from interviews and objective measurements are presented as means and measures of variation in Table 1.

**Table 1:** Overall Mean ($\overline{\mathrm{x}}$), standard deviation (s.d.) minimum (Min) and maximum (Max) values for Body Weight, Body Length, Heart Girth, BCS, Milk Yield and Price, for the three breed groups Red Maasai, Dorper and Crosses

Trait	Breed	Rank	$\overline{\mathrm{x}}$	s.d.	Min	Max
Body Weight (kg)	Red Maasai	Best	41.6	5.3	30.5	48.5
		Average	38.5	4.3	33.0	46.5
		Poor	32.4	4.5	24.5	40.0
	Dorper	Best	46.7	9.2	34.5	62.0
		Average	44.8	7.3	34.0	57.0
		Poor	37.0	3.9	30.5	45.0
	Cross	Best	43.3	7.8	30.5	62.0
		Average	39.2	5.5	30.5	53.5
		Poor	34.1	3.9	25.0	40.0
Body Length (cm)	Red Maasai	Best	62.9	7.3	55.0	83.0
		Average	61.4	6.4	54.0	82.0
		Poor	57.6	4.6	51.0	70.0
	Dorper	Best	64.0	8.0	55.0	83.0
		Average	64.8	6.4	54.0	77.0
		Poor	58.8	4.1	54.0	65.0
	Cross	Best	62.1	6.8	54.0	85.0
		Average	62.1	5.8	55.0	76.0
		Poor	58.5	8.6	31.0	77.0
Heart Girth (cm)	Red Maasai	Best	79.2	7.3	56.0	87.0
		Average	76.9	7.2	52.0	86.0
		Poor	72.9	4.7	63.0	80.0
	Dorper	Best	82.2	6.4	74.0	95.0
		Average	81.7	4.8	75.0	92.0
		Poor	76.2	3.7	70.0	81.0
	Cross	Best	80.8	6.0	72.0	91.0
		Average	76.8	6.6	56.5	89.0
		Poor	74.3	4.1	66.0	79.0
BCS (1–5)	Red Maasai	Best	3.03	0.6	1.5	4.0
		Average	2.47	0.7	1.5	3.5
		Poor	1.82	0.5	1.0	3.0
	Dorper	Best	2.85	0.7	1.5	4.0
		Average	3.04	0.8	2.0	4.5
		Poor	2.08	0.8	1.0	3.5
	Cross	Best	2.82	0.5	2.0	4.0
		Average	2.63	0.7	1.5	4.0
		Poor	2.00	0.6	1.0	3.0
Milk Yield^a^ (litre)	Red Maasai	Best	0.43	0.25	0.00	0.90
		Average	0.29	0.17	0.00	0.70
		Poor	0.16	0.15	0.00	0.53
	Dorper	Best	0.72	0.37	0.00	1.40
		Average	0.45	0.26	0.00	0.90
		Poor	0.21	0.14	0.00	0.41
	Cross	Best	0.53	0.21	0.00	0.90
		Average	0.35	0.17	0.00	0.70
		Poor	0.30	0.19	0.00	0.90
Price (KES)	Red Maasai	Best	5147	1344	3500	7000
		Average	4341	1229	3000	8000
		Poor	2988	718	2000	4500
	Dorper	Best	8462	4235	4000	20,000
		Average	7000	4439	3000	20,000
		Poor	4462	1785	2500	9000
	Cross	Best	5858	2353	3000	10,000
		Average	4532	1668	2000	8000
		Poor	3405	1166	1500	6000

Within each rank group there were 17 Red Maasai sheep, 13 Dorper and 19 Crosses (e.g. three of each Best, Average and Poor group), totally 147 ewes

^a^Minimum value 0.00 indicates no extra milk after feeding the lamb(s)

### Statistical analyses

All analyses were performed using the software “The R Project for Statistical Computing” (R Core Team 2013). Q-Q plots and histograms of residuals for all traits clearly resembled normal distributions. Therefore, linear models were used to analyse the records for all traits. The R package Companion to Applied Regression (Fox and Weisberg 2011) was used for the general linear model analyses and the package lsmeans (Lenth 2013) for estimation of the least squares means. Effects of age based on dentition and interactions between Breed and Rank and between Site and Rank were tested in preliminary analyses, but found to be non-significant and were therefore dropped in subsequent analyses.

The following fixed linear model, including the main effects and 2-way interactions that were significant, was finally used to explain the variation of the sheep traits recorded:

$$ {y}_{ijkl}=\mu +Sit{e}_i+Farme{r}_j\left( Sit{e}_i\right)+Bree{d}_k+Ran{k}_l+{\left( Breed\ast Site\right)}_{ki}+{e}_{ijkl} $$

where *y*_*ijkl*_ is the trait of interest, either Body Weight (kg), Body Length (cm), Heart Girth (cm), BCS (1–5), Milk Yield (litre) or Price (price if buying the ewe, in KES); *μ* is the overall mean for the trait; *Site*_*i*_ is the effect of the *i*th site (*i* = Amboseli, Isinya); *Farmer*_*j*_*(Site*_*i*_*)* is the effect of *j*th farmer nested within site *i* (*j =* 1*–*19); *Breed*_*k*_ is the effect of the *k*th breed group (*k =* Red Maasai, Dorper or Cross); *Rank*_*l*_ is the effect of the *l*th rank of the ewe (*l =* Best, Average or Poor); (Breed*Site)_*ki*_ is the interaction effect between *Breed*_*k*_ and *Site*_*i*_; and *e*_*ijkl*_ is the random residual effect.

### Ranking and weighting of traits

Every farmer gave their reasons for the ranking of the ewes. All reasons given were grouped into seven trait categories based on the most commonly appearing characteristics. The category of Body Size and Growth included reasons related to live weight as well as lamb and ewe growth rate, whereas Body Condition included reasons related to body fat cover. Milk Production was kept as a separate trait. Reproduction and Mothering Ability were grouped together and included reasons referring to twinning rate and lamb survival. Drought Tolerance and Disease Resistance were considered as separate traits. Breed Attributes included answers on colour, legs and hoofs and some specific features of each breed. Due to the open nature of questions presented to the farmers, their answers on reasons might in some cases have been transcribed and grouped differently.

Individual farmer’s responses of reasons were ordered by importance with the assumption that each farmer valuated the first, second and the third reasons approximately in the same manner with the same step length in-between the numbered reasons. The following equation, corrected from Bett et al. (2009b) was used to calculate the weighted reasons (WR*i*) of each trait (*i*) to be used for weighting of traits for each breed and site.

$$ W{R}_i={\displaystyle \sum_{j=1}^3{r}_j{X}_{ji}/\left({\displaystyle \sum_i}{\displaystyle \sum_{j=1}^3}{r}_j{X}_{ji}\right)} $$

where $Xji$ is the number of respondents giving Reason with order *j*, *j* = 1, 2, 3 to trait *i*, where *i* = Body Size and Growth, Condition, Milk Production, Reproduction and Mothering Ability, Drought Tolerance, Disease Resistance, or Breed Attributes. $r$_j_ is the weight corresponding to Reason *j*. The weight is given by *r*_*1*_ = 3, *r*_*2*_ = 2 and *r*_*3*_ = 1. The weights were arbitrarily chosen in the same way as in other studies (Bett et al. 2009a; Bett et al. 2009b; Mirkena et al. 2012; Mbuthia et al. 2014; Gebreyesus et al. 2013). Preliminary analyses were conducted with equal weightings for the three ordered reasons as well as with more differentiated weights, both analyses giving essentially the same results.

Ewes that were designated as being Average were used for farmers to find the midrange in their flocks but were not further analysed. Subsequent analyses were focused on the Best and Poor quality ewes within each breed group. Results for the Best ewes were interpreted as being the most preferred traits for each breed group from farmers’ assessments. Results for both the most preferred traits of the Best and the most inferior traits demonstrated in the Poor quality ewes were interpreted as indicators of the most important traits to improve for each breed and site. The results were used for the pure Red Maasai and Dorper breeds as basis for presenting weighted reasons of traits to be considered in a breeding objective for improvement of those breeds in Amboseli and Isinya. The reasons should be interpreted in the context of the present breeding practices where Dorper is used primarily for crossbreeding with Red Maasai. These weighted reasons, therefore, cannot be directly used as selection index weights for pure breeding. A regrouping of traits was also elaborated on to get an overview of the type of trait categories that characterize each breed by site.

## Results

### Descriptive statistics

Records and dentition showed that farmers selected ewes with ages ranging from 1 to 9 years and with an average age of 3.5 years. Among all animals studied at the two sites, ewes classified as Best had higher mean values for all traits (i.e. Body Weight, Body Length, Heart Girth, BCS, Milk Yield and Price) compared with the animals classified as Poor quality (Table 1). The ewes chosen as being Average generally had mean values that were in-between the best and the poor groups. Dorper had higher mean values than Red Maasai and Crosses for most of the traits studied.

### Effects of site and breed

There were significant effects of Site, Farmer within Site, Breed, Rank (Best, Average and Poor) and also for the interaction between Breed and Site for Body Weight and Price (Table 2). Rank and Farmer within Site significantly affected all traits. There were also highly significant differences between Site and Breed for Milk Yield.

**Table 2:** Levels of significance for effects of various factors in the model for the traits Body Weight, Body Length, Heart Girth, BCS, Milk Yield and Price

Factor	Body Weight	Body Length	Heart Girth	BCS	Milk Yield	Price
Site	***	***	*ns*	*ns*	***	***
Breed	***	*ns*	**	*ns*	***	***
Rank	***	***	***	***	***	***
Farmer(Site)	**	*	***	**	***	***
(Breed*Site)	*	*ns*	*	*ns*	*ns*	***

Significance levels, ***p < 0.001; **p < 0.01; *p < 0.05; *ns* non significant

Least-squares means for important traits are shown in Table 3 for each Breed within Site and for Rank categories across Sites. Values for Body Weight and Body Length were higher in Isinya for all breed groups. The Red Maasai ewes were 1.6 kg heavier in Isinya than in Amboseli, and Dorper ewes and Crosses weighed 5.9 and 6.7 kg more in Isinya, respectively. BCS and Milk Yield were slightly higher in Amboseli. The price the farmer was willing to pay for the sheep (Price) was higher for all breed groups in Isinya, with the largest price difference between sites for Dorper ewes. Red Maasai ewes were on average valued at 820 KES (22 %) more in Isinya compared to Amboseli, whereas Dorper and crossbred ewes in Isinya were regarded as having approximately twice the value of ewes of the same breed groups in Amboseli.

**Table 3:** Least squares means ± standard error for Body Weight (kg), Body Length (cm), Heart Girth (cm), BCS (1–5), Milk Yield (litre) and Price (KES) by levels of breed by site and for each rank across sites

Level	Body Weight	Body Length	Heart Girth	BCS	Milk Yield	Price
Amboseli						
Red Maasai	36.7 ± 0.93^ab^	58.7 ± 1.11^a^	77.1 ± 0.88^a^	2.9 ± 0.60^a^	0.4 ± 0.04^a^	3740 ± 136^ab^
Dorper	39.2 ± 1.12^a^	60.2 ± 1.35^a^	78.3 ± 1.06^a^	3.9 ± 0.72^b^	0.6 ± 0.05^b^	4090 ± 165^a^
Cross	35.7 ± 0.79^b^	57.7 ± 0.94^a^	75.5 ± 0.74^a^	2.6 ± 0.50^ab^	0.4 ± 0.04^ab^	3440 ± 116^b^
Isinya						
Red Maasai	38.3 ± 0.98^a^	62.6 ± 1.24^a^	75.6 ± 1.61^a^	2.3 ± 0.25^a^	0.2 ± 0.03^a^	4560 ± 382^a^
Dorper	45.1 ± 1.17^b^	63.2 ± 1.48^a^	78.6 ± 1.92^a^	3.0 ± 0.30^a^	0.4 ± 0.04^b^	8460 ± 456^b^
Cross	42.4 ± 0.98^b^	64.4 ± 1.24^a^	79.1 ± 1.61^a^	2.4 ± 0.25^a^	0.4 ± 0.03^b^	5890 ± 382^c^
Overall rank						
Best	43.7 ± 0.70^a^	62.8 ± 0.85^a^	80.9 ± 0.74^a^	2.9 ± 0.09^a^	0.6 ± 0.02^a^	6340 ± 221^a^
Average	40.6 ± 0.70^b^	62.4 ± 0.85^a^	78.4 ± 0.74^b^	2.7 ± 0.09^b^	0.4 ± 0.02^b^	5160 ± 221^b^
Poor	34.4 ± 0.70^c^	58.2 ± 0.85^b^	74.5 ± 0.74^c^	1.9 ± 0.09^c^	0.2 ± 0.02^c^	3580 ± 221^c^

^a,b,c^Means within column with different superscripts are significantly different at *p* < 0.05

1 USD = 84 KES

Farmers’ choices of Best, Average and Poor quality ewes were confirmed by the objective measurements (Table 3). Animals chosen as Best were on average 3.1 kg heavier than the Average ewes and 9.3 kg heavier than the Poor quality ewes. BCS did not differ significantly between the Best and Average ewes, but was much lower for the Poor quality ewes. Milk Yield was highest among the Best ewes and lowest among the Poor quality ewes. Farmers expressed willingness to pay almost twice as much for ewes classified as being Best compared to the Poor quality ewes and to pay 23 % more for the Best ewes than for an Average ewe.

### Weighted reasons for farmers’ choices of ewes

**Fig. 1:**
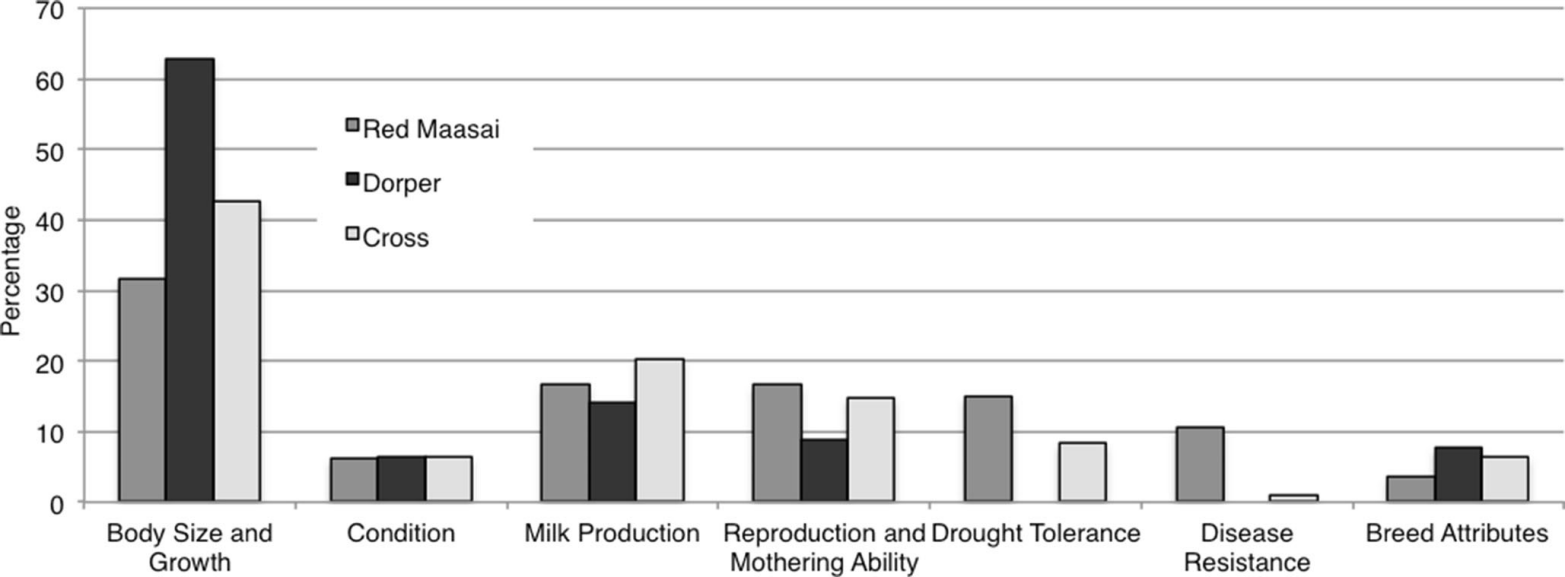
Weighted reasons for farmers classifying ewes as Best quality across sites: relative importance of trait preferences for each breed group in percent

For the Best ewes across both sites, the weighted reasons for appreciation of these animals in the flock are presented in Fig. 1. Body Size and Growth were considered as the most important traits irrespective of breed, though most clearly for Dorper. Milk Production was ranked as the second most important trait regardless of breed. Red Maasai was slightly more appreciated for its good Reproduction and Mothering Ability and was also more appreciated than the other breed groups for its Drought Tolerance and Disease Resistance. It is noteworthy that no Dorper ewes were mentioned to have or excel in these two adaptive traits.

**Fig. 2:**
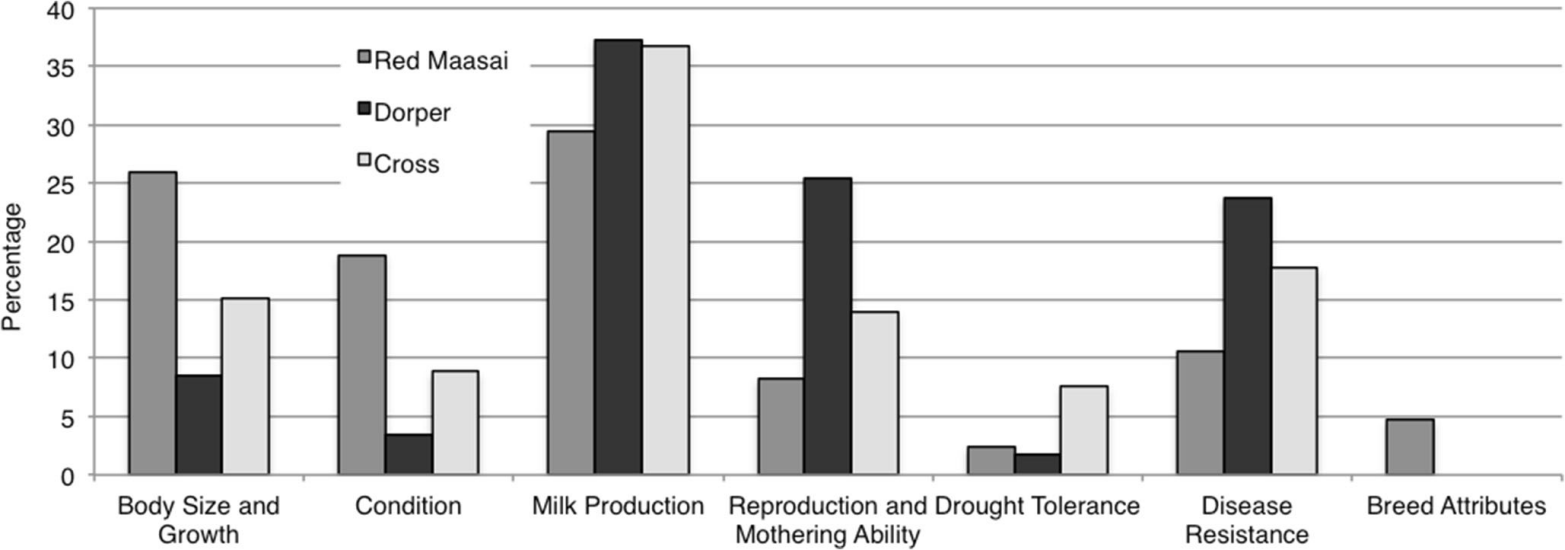
Weighted reasons for farmers classifying ewes as Poor quality across sites: relative importance of trait groups identified as inferior for each breed group in percent

For ewes considered to be Poor quality ewes, the weighted reasons are shown in Fig. 2. For all breed groups, low Milk Production was most often cited as the reason for a ewe being classified as of Poor quality. For Red Maasai, small Body Size and Growth and poor Condition were commonly cited. For Dorper, poor Reproduction and Mothering Ability and Disease Resistance were often cited as a reason for the ewe being categorized as a Poor quality ewe.

### Weighted reasons by breed and site

Trait preferences for each trait group by breed within site for Best ewes are shown in Table 4. The Best ewes, across breeds and sites, were most preferred for their Body Size and Growth. However, the weighted reasons were consistently higher in Isinya than in Amboseli for Body Size and Growth; 0.35 vs. 0.27 for Red Maasai, 0.71 vs. 0.53 for Dorper and 0.58 vs. 0.30 for Crosses. For Red Maasai in Amboseli, Reproduction and Mothering Ability were equally as important as Body Size and Growth. Milk Production was considered as second or third most important trait for all breed groups across sites. Condition was mentioned for the Best ewes across breeds in Amboseli but not in Isinya. Notably in Isinya, farmers mentioned the Best ewes of Red Maasai because of their Drought Tolerance and Disease Resistance, whereas hardly any Dorper and Crosses were mentioned for these traits at any of the sites.

**Table 4:** Trait preferences as assessed by farmers for the Best quality ewes

	Amboseli					Isinya				
	Reasons^a^				Weighted reasons^b^	Reasons^a^				Weighted reasons^b^
Breed and Trait group	1	2	3	Sum		1	2	3	Sum	
Red Maasai										
Body Size and Growth	6	4	3	13	0.27	12	8	3	23	0.35
Condition	0	4	3	7	0.15	0	0	0	0	0.00
Milk Production	3	4	1	8	0.17	3	4	4	11	0.17
Reproduction and Mothering Ability	9	4	0	13	0.27	0	4	2	6	0.09
Drought Tolerance	6	0	0	6	0.13	9	2	0	11	0.17
Disease Resistance	0	0	0	0	0.00	3	8	1	12	0.18
Breed Attributes	0	0	1	1	0.02	3	0	0	3	0.05
Dorper										
Body Size and Growth	12	6	1	19	0.53	18	10	2	30	0.71
Condition	3	2	0	5	0.14	0	0	0	0	0.00
Milk Production	3	0	2	5	0.14	0	2	4	6	0.14
Reproduction and Mothering Ability	0	2	0	2	0.06	3	2	0	5	0.12
Drought Tolerance	0	0	0	0	0.00	0	0	0	0	0.00
Disease Resistance	0	0	0	0	0.00	0	0	0	0	0.00
Breed Attributes	0	2	3	5	0.14	0	0	1	1	0.02
Cross										
Body Size and Growth	12	2	4	18	0.30	21	4	3	28	0.58
Condition	3	4	0	7	0.12	0	0	0	0	0.00
Milk Production	3	6	3	12	0.20	0	8	2	10	0.21
Reproduction and Mothering Ability	6	4	0	10	0.17	0	4	2	6	0.13
Drought Tolerance	3	2	1	6	0.10	3	0	0	3	0.06
Disease Resistance	0	0	0	0	0.00	0	0	1	1	0.02
Breed Attributes	3	2	2	7	0.12	0	0	0	0	0.00

^a^Reasons 1–3, where the most important reason was weighted by 3, etc.

^b^Weighted reasons as proportion of the total sum of reasons by breed and site

Weighted reasons for farmers classifying ewes as a Poor quality ewe are shown in Table 5, by breed and site. In Isinya, low Milk Production was a primary reason in all breed groups for classifying a ewe as poor. For Red Maasai, low Body Size and Growth was a major reason. For Dorper and Crosses, across sites, insufficient Reproduction and Mothering Ability as well as lack of Disease Resistance were given as the major reasons.

**Table 5:** Traits identified as inferior by farmers for the Poor quality ewes

	Amboseli					Isinya				
	Reasons^a^				Weighted reasons^b^	Reasons^a^				Weighted reasons^b^
Breed and Trait group	1	2	3	Sum		1	2	3	Sum	
Red Maasai										
Body Size and Growth	3	0	3	6	0.15	9	2	5	16	0.36
Condition	9	2	2	13	0.33	3	0	0	3	0.07
Milk Production	3	6	0	9	0.23	6	8	2	16	0.36
Reproduction and Mothering Ability	0	6	1	7	0.18	0	0	0	0	0.00
Drought Tolerance	0	0	0	0	0.00	0	2	0	2	0.04
Disease Resistance	3	0	0	3	0.08	6	0	0	6	0.13
Breed Attributes	0	2	0	2	0.05	0	2	0	2	0.04
Dorper										
Body Size and Growth	3	0	0	3	0.09	0	0	2	2	0.07
Condition	0	0	2	2	0.06	0	0	0	0	0.00
Milk Production	0	6	2	8	0.25	6	6	2	14	0.52
Reproduction and Mothering Ability	6	4	0	10	0.31	3	2	0	5	0.19
Drought Tolerance	0	0	0	0	0.00	0	0	1	1	0.04
Disease Resistance	9	0	0	9	0.28	3	2	0	5	0.19
Breed Attributes	0	0	0	0	0.00	0	0	0	0	0.00
Cross										
Body Size and Growth	3	0	2	5	0.12	0	4	3	7	0.18
Condition	0	6	1	7	0.17	0	0	0	0	0.00
Milk Production	3	4	4	11	0.27	15	0	3	18	0.47
Reproduction and Mothering Ability	3	2	2	7	0.17	0	2	2	4	0.11
Drought Tolerance	3	0	1	4	0.10	0	2	0	2	0.05
Disease Resistance	3	4	0	7	0.17	6	0	1	7	0.18
Breed Attributes	0	0	0	0	0.00	0	0	0	0	0.00

^a^Reasons 1–3, where the most important reason by 3, etc.

^b^Weighted reasons as proportion of the total sum of reasons by breed and site

To show the relative importance of trait categories and to compare the purebreds and also the sites, the weighted reasons for the Best and the Poor quality ewes were summarized by further clustering related traits (Table 6). For this purpose, Body Size and Growth and Condition were considered as before, whereas Milk Production was grouped together with Reproduction and Mothering Ability (Reproduction and Milk). The adaptive traits Drought Tolerance and Disease Resistance were grouped together and referred to as Adaptation, whereas Breed Attributes was excluded due to low frequencies and diversity of responses. It could then be stated that Reproduction and Milk as well as Body Size and Growth stand out as the generally most important trait category across both breeds and sites.

**Table 6:** Relative importance (accumulated weighted reasons) of trait categories as assessed by farmers for Best and Poor quality ewes by breed and site

	Best quality				Poor quality			
	Red Maasai		Dorper		Red Maasai		Dorper	
Trait complex	Amboseli	Isinya	Amboseli	Isinya	Amboseli	Isinya	Amboseli	Isinya
Body size and Growth	0.28	0.37	0.61	0.73	0.16	0.37	0.09	0.07
Condition	0.15	0.00	0.16	0.00	0.34	0.07	0.06	0.00
Reproduction and Milk	0.45	0.27	0.23	0.27	0.42	0.37	0.56	0.70
Adaptation	0.13	0.37	0.00	0.00	0.08	0.19	0.28	0.22

When ranking all ewes per farm across breed groups, the farmers in Amboseli ranked the Red Maasai and Dorper breeds as being equally good. The inferior adaptive traits of Dorper ewes were compensated by their better Body Size and Growth. In Isinya, however, farmers clearly preferred Dorper ewes (Table 7).

**Table 7:** Average rank of ewes (scale 1–9, 1 = best, 9 = worst) across breed groups by site among all the studied ewes

		Average rank		
Site	No. of ewes	Red Maasai	Dorper	Crosses
Amboseli	72	4.9	4.8	5.5
Isinya	75	5.7	4.2	5.0

## Discussion

Regular interactions throughout the sheep recording project with the pastoral communities within the two sites indicated that the number of purebred sheep is low compared with the number of crosses between Red Maasai and Dorper at different “up-grading” levels. This was confirmed when searching for farms having all three breed groups and in large enough numbers. It was difficult to find such farms. Although no organized breeding programme is in place, farmers are, however, very knowledgeable as far as their own flocks are concerned and select breeding stock based on own memory and values of the animal. For a sustainable use of existing sheep genetic resources in the targeted area, it is important to design breeding strategies both for conservation and genetic improvement in accordance with farmers’ preferences in addition to supporting a sustainable crossbreeding system. As a first step in designing a relevant breeding programme, the results of this study are essential, as they indicate important traits to be included in the breeding objectives based on farmers’ own evaluation of strengths and weaknesses of their sheep.

### Study sites and farmer participation

The selected study sites are representative of production systems where the Red Maasai and Dorper sheep and their crosses are currently being reared within Kenya. There are, however, large differences in the environment and production systems between the Isinya and Amboseli sites even though they are geographically close to each other. This was also noted by Liljestrand (2012).

When assessing breeding objectives and designing breeding programmes, it is important to actively involve the farmers in the whole process (Mueller et al. 2015). This will ensure that the farmers’ needs are taken into account and that are they provided with the support needed for the breeding programme to function (Philipsson et al. 2011). Pedigree and performance recording are prerequisites for any genetic improvement programme development and implementation. Prior to this study, a basic recording scheme had been adopted. In the recording scheme, farmers recorded the identity, live weight at different ages, body measurements, medical treatments and lambing information of the individual animals. To increase recorded information, additional measurements of BCS were taken and farmers provided their own assessment of their animals based on their experiences. Building on the on-going recording system, it was easier for the farmers to respond to the additional research questions in our study. The farmers’ subjective ranking of animals as Best, Average or Poor quality was supported by the objective measures of body weight, linear measurements of conformation and BCS (Tables 1 and 3). There could be some uncertainty about the accuracy of the farmers’ recall at the time of the interview about how much milk the ewes were producing. No empirical milk production records over an extended period of time were available. The response given on the price the farmer is willing to pay for the ewe if it was bought at the market to be used for breeding purposes gave an indication about the value of the animal as a breeding ewe, although this is a simplification of the ewe market. Preferably, more objective measures of the market value of animals would include prices of young animals for slaughter.

From the subset of the populations sampled, distinct differences between breeds, rank and site were noted for a number of traits, especially regarding Body Weight. We believe that by prompting farmers to rank their own ewes enabled them to give more objective and accurate information, than if the farmers would have ranked, to them unknown animals, or if data were gathered only by surveys without animals available.

The reasons mentioned for the selection of the Best quality ewes were intended to reflect what traits of each breed group were appreciated by the farmers and if such preferences depended on breed type. Clear breed differences and interactions with site were observed. The reasons given for sets of preferred, or inferior traits in Poor quality ewes, were done in accordance with the farmers’ ordering of the traits. Similar use of weighted reasons of traits in livestock populations to provide information for developing breeding objectives has been carried out by Bett et al. (2009a, b), Mirkena et al. (2012) and Gebreyesus et al. (2013).

Farmers, in this study, provided three main reasons for ranking a specific ewe as of Best, Average or Poor quality. There may have been other reasons beyond the top three; however, these were not taken into account in this study. Answers from farmers might in some cases also have been grouped differently giving opportunities for alternative interpretations or because the traits are closely related. For example, there could be a close relationship between the various measures of Body Size and body Condition, however, the relationship between body size and growth rate was greater. Milk Production could also be grouped together with the related Reproduction and Mothering Ability trait complexes. Lamb survival, included in the trait category of Reproduction and Mothering Ability, is dependent on its dam’s milk production, which is part of the maternal effects, but lamb survival is also influenced directly by various environmental factors and the genes of the lamb. Drought Tolerance and Disease Resistance may also be grouped together as these traits are likely to have co-evolved together over time and generations for a given breed, as part of the adaptation to environmental challenges and stresses such as parasites, high temperatures and low feed and water availability.

### Interaction of breed with site

In Amboseli, there were only slight breed differences in Body Weights between breed-types, whereas in Isinya Dorper and Crosses were superior to Red Maasai. The interaction between site and breed is demonstrated in Table 3, which points at a better adaptability of the Red Maasai to harsher climatic conditions. Significant breed by site interactions regarding live weight for Red Maasai and Dorper was also observed by Okeyo and Baker (2005). Adaptability of an indigenous livestock breed such as Red Maasai, to a challenging environment, is expected due to what has been reported in other studies (e.g. Preston and Allonby 1979; Osaer et al. 1999; Mirkena et al. 2010).

Some breeds may be more commercially suitable in regions where, not only the environment, but also infrastructure and distance to market favour them (Marshall 2014). Isinya has the advantage of being located closer to large towns, and the farmers were more market oriented. Prices for animals were higher and the farmers were willing to pay more for larger sheep with good growth potential (Tables 1 and 3). In Amboseli, on the other hand, the infrastructure is less developed and farmers depend on livestock to provide for their daily livelihoods. Condition and survival traits of the animals were therefore more highly valuable in Amboseli.

The interaction of preferred breed by site is supported by the results shown in Table 7. Farmers’ ranking of preferred ewes across breed groups was used to calculate the mean ranking for ewes of each breed and site. The Dorper was preferred over the Red Maasai in Isinya, whereas in Amboseli both breeds were equally preferred, although for different reasons. Hence, the farmers continue to strive to retain Red Maasai through some form of purebreeding and crossbreeding.

### Preferred traits for a breeding objective

The information on reasons why ewes were classified as of Best quality shows that increased Body Size and Growth was preferred in all three breed groups at both sites (Fig. 1; Table 4), but it was more pronounced for Dorper and Crosses and more markedly in Isinya. The desire for larger animals in pastoralists’ flocks has also been expressed in previous studies (Kiriro 1994; Kosgey et al. 2008; Kariuki et al. 2010). When analysing the information on what traits were considered as inferior, i.e. that contributed to the classification of ewes as of Poor quality, Milk Production turned out to be a surprisingly important trait at both sites (Fig. 2). It was said to have increased in importance after the massive deaths of cattle during the severe droughts in 2008–2009 (Liljestrand 2012; Audho et al. 2015). Milk Production was highly ranked for all three breed groups (Table 5) and especially emphasized for Dorper.

Red Maasai ewes were appreciated for their reproduction and adaptive traits. In Dorper and Crosses, however, Reproduction and Mothering Ability were emphasized as inferior traits in addition to lack of Disease Resistance, despite the fact that no severe drought occurred around the time of data collection.

The summarised accumulated weighted reasons for the four clustered trait categories ‘Body Size and Growth’, ‘Condition’, ‘Reproduction and Milk’ and ‘Adaptation’ show the importance of giving high weights in the breeding objective both to Body Size and Growth and to Reproduction and Milk production for both breeds across sites (Table 6). In Amboseli, Condition is also a highly weighted trait for both breeds. Traits constituting Adaptation are especially important to improve in the Dorper breed.

### Aspects on potential breeding strategies

When designing breeding strategies, it is important to develop the breeding programmes in full awareness of the differences in trait preferences found for the two sites and also to consider the extent to which the genetic variation within breeds allows the use of the two sheep breeds across sites. It was evident that both Red Maasai and Dorper sheep have advantages as well as weaknesses, and that both breeds ought to be improved through within-breed selection. Performance of the progeny can also be improved by crossbreeding between these breeds, especially in Isinya. It is, therefore, important that Red Maasai sheep will be continuously improved and conserved and used in purebreeding as well as in strategic crossbreeding programmes.
